# Mapping the global clinical landscape of NK cell therapies for solid tumors: an analysis based on the ClinicalTrials.gov for the 2005–2024 period

**DOI:** 10.1007/s00432-025-06329-0

**Published:** 2025-10-04

**Authors:** Wenjuan Wei, Ximeng Wu, Lizhi Wang, Jianing Zhang, Chan Zhang, Daqing Wang

**Affiliations:** Liaoning Provincial Key Laboratory for Early Diagnosis and Biotherapy of Malignant Tumors in Children and Women, Dalian Women and Children’s Medical Center (Group), Dalian, China

Solid tumors account for over 90% of all malignant cancers globally. Their characteristics such as heterogeneity, immunosuppressive microenvironment, and physical barriers formed by a dense extracellular matrix, make them significantly more difficult to treat than hematological tumors. Natural killer (NK) cells, can exert anti-cancer effects without prior activation or antigen presentation. This diversity offers broad application prospects for NK cells in tumor therapy (Vivier et al. 2024), (Eckstrom et al. 2025). However, comprehensive data on the global clinical landscape of this area remains limited. To address this, clinical studies on NK cell therapies for solid tumors were searched within the ClinicalTrials.gov database. We used a search methodology including the terms (Condition/disease: Tumor; Intervention/treatment: NK cells) AND Study Status: All studies. As of December 31, 2024, a total of 784 trials were initially screened out, and 141 trials were selected after manual screening for relevance to solid tumors.

For the geographical distribution of these trials (Fig. 1A), China ranked first among all 14 countries and regions, contributing 62 trials (44.0%), followed by the United States with 45 trials (31.9%), which led in North America. Spain ranked highest among European countries with 4 trials (2.8%). It is evident that Asian countries accounted for the majority of all trials (87, 61.7%), while no relevant trials were conducted in Africa or South America. In the future, narrowing these gaps through international cooperation will help promote more equitable access to treatment and research.

**Fig. 1:**
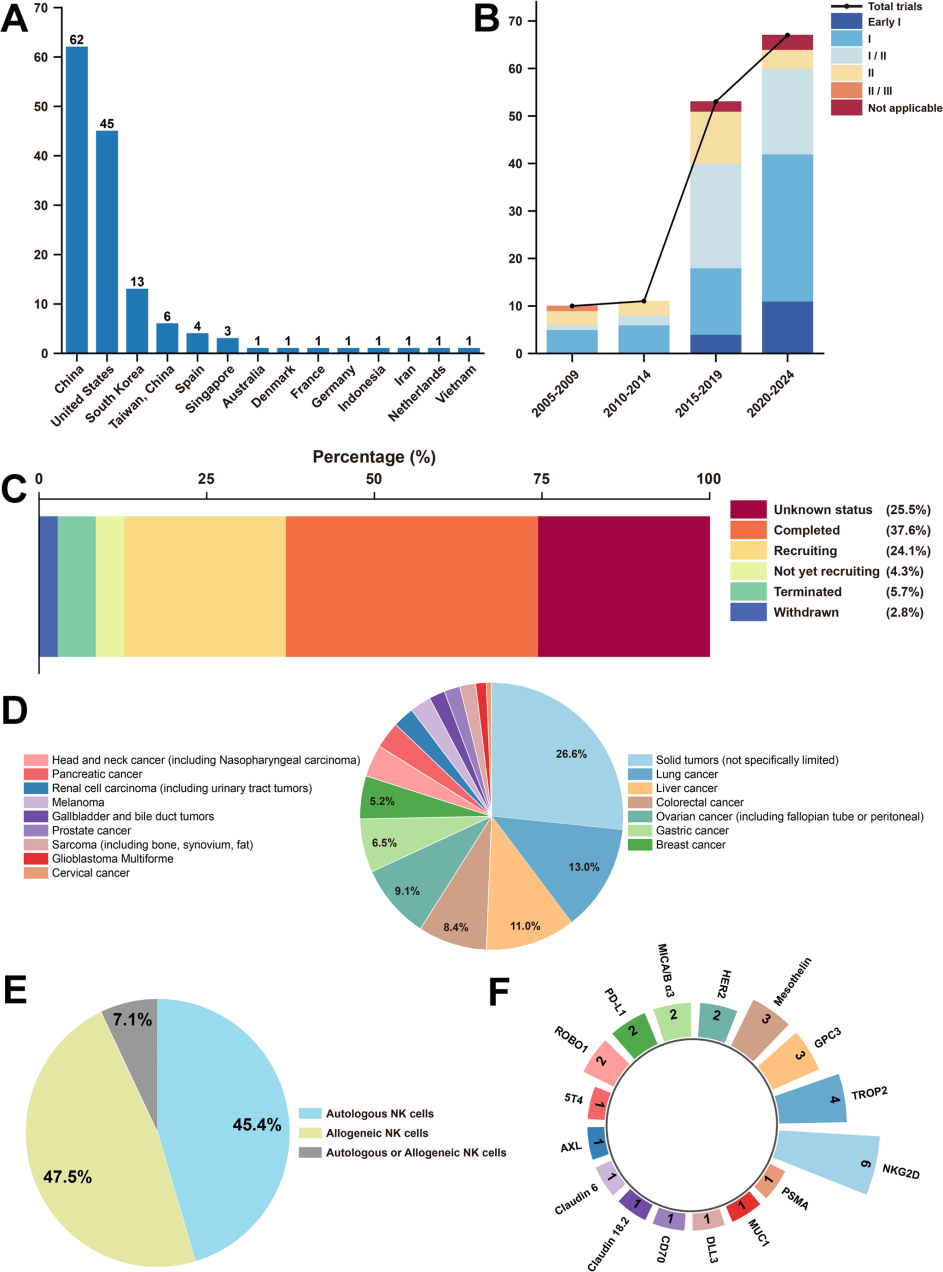
Global landscape of NK cell therapy trials for solid tumors. (A) Number of clinical trials in all the 14 countries and regions. (B) The number of clinical trials worldwide from 2005 to 2024, divided into five-year periods. Each period includes the total number of trials and a breakdown by phase. (C) NK cell therapy trials, by status, as of December 31, 2024. (D) Types of solid tumors treated with NK cells. (E) The source of NK cells for treatment. (F) Targets of CAR-NK cells for solid tumor treatment

From 2005 to 2014, the number of trials remained relatively low. However, a substantial rise was observed after 2015 (Fig. 1B). Phase I clinical trials constituted the largest proportion (56, 39.7%), followed by Phase I/II trials (43, 30.5%). Nevertheless, no Phase III or IV clinical trials have been initiated. These findings indicate that most clinical research remains in the early stages, primarily focusing on evaluating the safety and determining the appropriate dosage of NK cell applications.

Figure 1C shows the status of these trials, with a considerable portion already completed (53, 37.6%). These are followed by trials listed as unknown status (36, 25.5%) and those that are currently recruiting (34, 24.1%). A small proportion of trials were terminated (8, 5.7%) or withdrawn (4, 2.8%), with reasons including poor enrollment, medical disputes, changes in care settings, and business-related decisions.

Among the 141 clinical trials, the most widely targeted cancer type is lung cancer (20, 14.2%) (Fig. 1D). In addition, a large portion of the trials focused on solid tumors that are not limited to a specific type (41, 29.1%). These studies explore the use of different sources of NK cells, which are categorized into allogeneic NK cells (67, 47.5%) and autologous NK cells (64, 45.4%). The applications of these two types are nearly equivalent (Fig. 1E). Meanwhile, a small number of studies do not specify the source of NK cells (10, 7.1%).

The vast majority of autologous NK cells are derived from the patient’s peripheral blood. Induced pluripotent stem cells (iPSCs) from patients can also serve as a source of NK cells (NCT06245018, NCT05069935) which offer high homogeneity and quality control, genetic engineering flexibility, and inexhaustible cell source (Wei et al. 2025). Allogeneic NK cells are mainly obtained from umbilical cord blood and other donors, with only one case using NK cells derived from placental hematopoietic stem/progenitor cells (NCT05207722).

Furthermore, novel conceptual and functional NK cell platforms are emerging, such as metabolic remodeling natural killer cells (METR-NK cells) (NCT06395844), cytokine-induced memory-like natural killer cells (CIML NK cells) (NCT06318871, NCT06321484), transforming growth factor beta-imprinted natural killer cells (TGFβi NKs) (NCT06026657), and ACC, also known as antibody-cell conjugate, (ACE1702) (NCT04319757) (Li et al. 2021). These innovative features make them attractive cell types for the development of novel adoptive cellular immunotherapy strategies. In terms of dosage, the minimum administered dose of NK cells is 5.0 × 10^6^ cells/dose (NCT05845502), while the maximum reaches 12 × 10^9^ cells/dose (NCT06098898). Regarding combination therapy, NK cells are being investigated in conjunction with more than ten targeted agents or chemotherapy drugs, and can also be combined with other immune cells, such as dendritic cells (DCs) (NCT03815084) and γδT cells (NCT04990063).

CAR-NK cell therapy, as a novel form of immunotherapy, utilizes genetic engineering techniques to introduce specific chimeric antigen receptors (CARs) into NK cells (Gong et al. 2021), (Xie et al. 2020). In the treatment of solid tumors, 30 projects (21.3%) have employed CAR-NK technology, targeting 16 distinct antigens (Table S1). Among these, NKG2D was the most frequently selected target (6, 20.0%), followed by Trop2 (4, 13.3%) (Fig. 1F). As research into these targets continues to deepen, NK cell therapy is expected to become an integral component of comprehensive cancer treatment within the next 5 to 10 years (Peng et al. 2024).

In brief, NK cells possess the fundamental characteristics of an “off-the-shelf” therapeutic, providing new hope for patients and new directions for clinical research. Current research mainly focuses on enhancing their tumor-homing ability, cytotoxicity, and persistence. Strategies include the combination of cytokines, CAR modification, and the use of immune checkpoint inhibitors. Nevertheless, major challenges remain, such as immunosuppression within the solid tumor microenvironment and the short in vivo lifespan of NK cells. Further breakthroughs are needed to overcome these technical bottlenecks, improve efficacy and safety, and advance clinical translation.

## Supplementary Information

Below is the link to the electronic supplementary material.

Supplementary Material 1

## Data Availability

No datasets were generated or analysed during the current study.
