# Supplementary material for: Mapping the global clinical landscape of NK cell therapies for solid tumors: an analysis based on the ClinicalTrials.gov for the 2005–2024 period
Source: J Cancer Res Clin Oncol. 2025 Oct 4;151(10):277. doi: 10.1007/s00432-025-06329-0 (PMC12494998; doi:10.1007/s00432-025-06329-0)
Supplement: Supplementary file 1 — Supplementary Material 1 [file 432_2025_6329_MOESM1_ESM.docx]

**Table S1. CAR-NK** **cell therapies for solid tumors** **on the ClinicalTrials.gov from 2005 to 2024**

| **ID Number** | **Targets** | **Conditions** | **Phase** | **Status** | **Cell source** | **Regions** |
| --- | --- | --- | --- | --- | --- | --- |
| NCT03415100 | NKG2D | Metastatic Solid Tumors | I | Unknown status | Autologous or allogeneic | China |
| NCT05213195 | NKG2D | Refractory Metastatic Colorectal Cancer | I | Recruiting | Allogeneic | China |
| NCT06478459 | NKG2D | Advanced Pancreatic Cancer | Early I | Recruiting | Autologous | China |
| NCT05776355 | NKG2D | Ovarian Cancer | Not Applicable | Unknown status | Autologous | China |
| NCT06503497 | NKG2D | Pancreatic Cancer | Early I | Recruiting | Autologous or allogeneic | China |
| NCT05248048 | NKG2D | Refractory Metastatic Colorectal Cancer | Early I | Unknown status | Allogeneic | China |
| NCT06454890 | TROP2 | Relapsed/Refractory Non-Small Cell Lung Cancer | I/II | Not yet recruiting | Autologous or allogeneic | China |
| NCT06066424 | TROP2 | Advanced Solid Tumors | I | Recruiting | Allogeneic | United States |
| NCT05922930 | TROP2 | Platinum Resistant Ovarian Cancer, Mesonephric-like Adenocarcinoma, and Pancreatic Cancer | I/II | Recruiting | Allogeneic | United States |
| NCT06358430 | TROP2 | Colorectal Cancer, Minimal Residual Disease | I | Recruiting | Allogeneic | United States |
| NCT06652243 | GPC3 | Hepatocellular Carcinoma | Early I | Recruiting | Allogeneic | China |

**Table S1** continued

| **ID Number** | **Targets** | **Conditions** | **Phase** | **Status** | **Cell source** | **Regions** |
| --- | --- | --- | --- | --- | --- | --- |
| NCT05845502 | GPC3 | Advanced Hepatocellular | Not Applicable | Terminated | Autologous | China |
| NCT06652243 | GPC3 | Hepatocellular Carcinoma | Early I | Recruiting | Allogeneic | China |
| NCT03692637 | Mesothelin | Epithelial Ovarian Cancer | Early I | Unknown status | Autologous | China |
| NCT05686720 | Mesothelin | Advanced Triple Negative Breast Cancer | Early I | Unknown status | Allogeneic | China |
| NCT05410717 | Mesothelin, GPC3, Claudin 6, or AXL | Advanced solid tumors | I | Recruiting | Autologous | China |
| NCT06341647 | HER2 | Breast Cancer, Gastric Cancer, Gastroesophageal Junction Adenocarcinoma | I | Withdrawn | Allogeneic | South Korea |
| NCT04319757 | HER2 | Advanced or Metastatic HER2-expressing Solid Tumors | I | Completed | Allogeneic | United States |
| NCT06342986 | MICA/B α3 | Recurrent Ovarian, Fallopian Tube, and Primary Peritoneal Cancer | I | Recruiting | Allogeneic | United States |
| NCT05395052 | MICA/B α3 | Advanced Solid Tumors | I | Terminated | Allogeneic | United States |

**Table S1** continued

| **ID Number** | **Targets** | **Conditions** | **Phase** | **Status** | **Cell source** | **Regions** |
| --- | --- | --- | --- | --- | --- | --- |
| NCT04847466 | PD-L1 | Recurrent/Metastatic Gastric or Head and Neck Cancer | II | Active, not recruiting | Allogeneic | United States |
| NCT04050709 | PD-L1 | Locally Advanced or Metastatic Solid Cancers | I | Active, not recruiting | Allogeneic | United States |
| NCT03940820 | ROBO1 | Solid Tumors | I/II | Unknown status | Autologous or allogeneic | China |
| NCT03941457 | ROBO1 | Pancreatic Cancer | I/II | Unknown status | Autologous | China |
| NCT05194709 | 5T4 | Advanced Solid Tumors | Early I | Unknown status | Allogeneic | China |
| NCT05703854 | CD70 | Advanced Renal Cell Carcinoma, Mesothelioma and Osteosarcoma | I/II | Recruiting | Allogeneic | United States |
| NCT06464965 | Claudin 18.2 | Gastric Cancer, Pancreatic Cancer | I | Recruiting | Allogeneic | China |
| NCT05507593 | DLL3 | Extensive Stage Small Cell Lung Cancer | I | Unknown status | Autologous or allogeneic | China |
| NCT02839954 | MUC1 | MUC1 Positive Relapsed or Refractory Solid Tumor | I/II | Unknown status | Autologous | China |
| NCT03692663 | PSMA | Metastatic Castration-Resistant Prostate Cancer | Early I | Unknown status | Autologous | China |
